# Supplementary material for: Established Pseudomonas syringae pv. tomato infection disrupts immigration of leaf surface bacteria to the apoplast
Source: Front Microbiol. 2025 Feb 3;16:1546411. doi: 10.3389/fmicb.2025.1546411 (PMC11830748; doi:10.3389/fmicb.2025.1546411)
Supplement: Supplementary file 1 [file Data_Sheet_1.PDF]

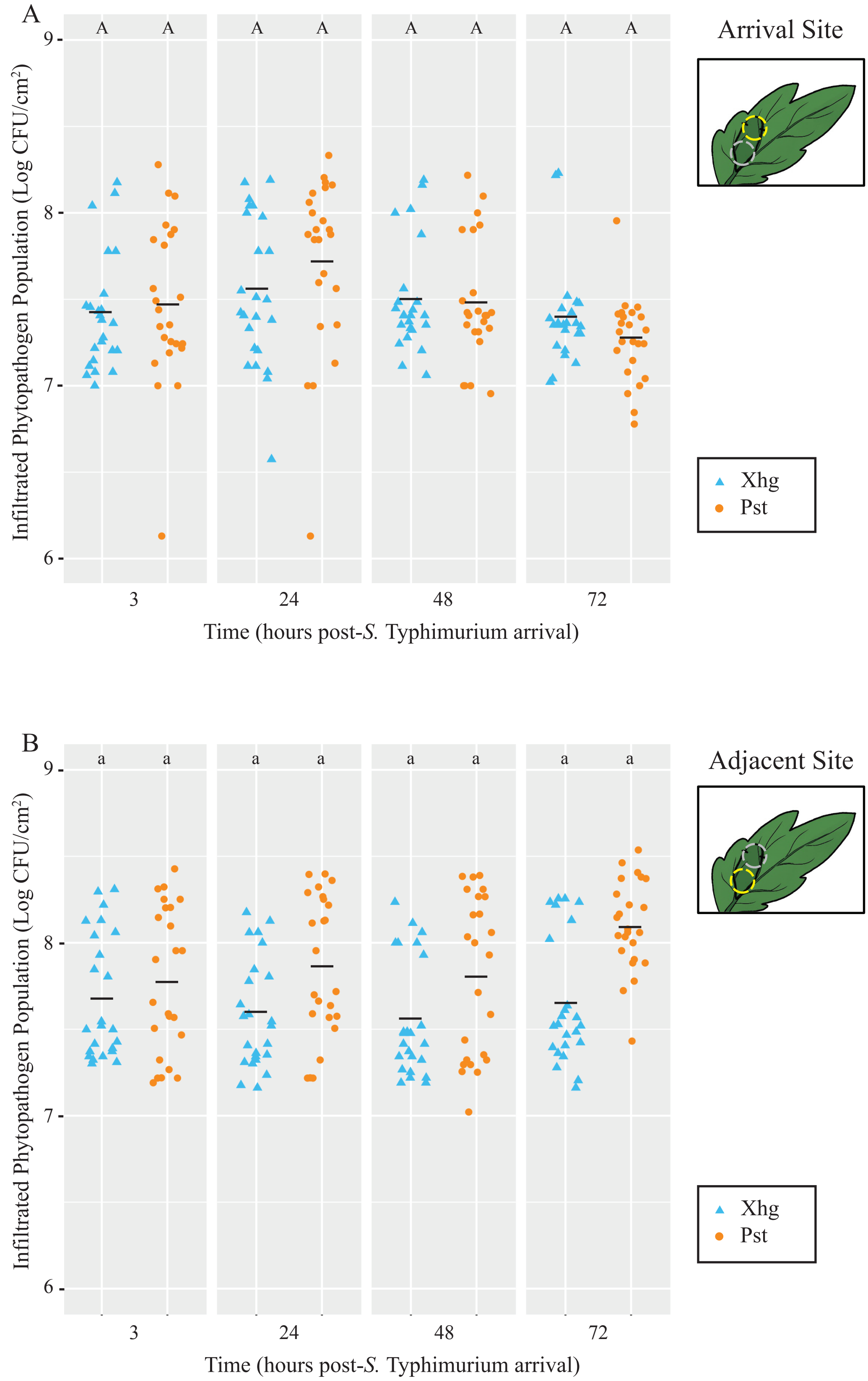

Figure S1. Infiltrated phytopathogen populations remain stable throughout the *S.* Typhimurium arrival experiment. Infiltrated Xhg (cyan triangles) and Pst (orange circles) populations were monitored 3, 24, 48, or 72 hours after *S.* Typhimurium arrival on tomato leaves. Leaves were sampled at the arrival site (A) and a distinct adjacent site within the infiltrated area (B). Data from three independent experiments are presented as log CFU/cm^2, and each symbol represents bacterial populations from one tomato leaf. Half of the leaves from each treatment and time point were treated with UV irradiation but data were collapsed as there was no significant difference between UV-treated and non-UV-treated samples ($P>0.01$). Means for each treatment at each time point are depicted with horizontal black lines. Letters denote significant differences between treatments within a single time point and leaf site ($P<0.05$). Combining three independent experiments, n=24 leaves per treatment per time point.
